# Enhancing Relaxation Through Co-Design with People Living with Dementia: The CALM Robot

**DOI:** 10.1093/geroni/igaf122.3243

**Published:** 2025-12-31

**Authors:** Lillian Hung, Irene Chen, Harleen Hundal, Lynn Jackson, Albin Soni, Jason Fu

**Affiliations:** The University of British Columbia, Vancouver, British Columbia, Canada; The University of British Columbia, Vancouver, British Columbia, Canada; The University of British Columbia, Vancouver, British Columbia, Canada; The University of British Columbia, Vancouver, British Columbia, Canada; The University of British Columbia, Vancouver, British Columbia, Canada; The University of British Columbia, Vancouver, British Columbia, Canada

## Abstract

The involvement of individuals living with dementia in the co-design of assistive technologies is crucial to ensuring that these innovations meet their needs, preferences, and lived experiences. This study presents insights from a co-design process involving 4 people living with dementia (PLwD), 6 caregivers, and 2 healthcare professionals to develop a social robot designed to facilitate relaxation and deep-breathing exercises. The research employed a co-design framework, integrating PLwD as active participants in design workshops, prototype evaluations, and reflection meetings. Qualitative data were collected through structured discussions, observation notes, and participant feedback. The iterative design process allowed for continuous refinement of the robot’s features, focusing on tactile engagement, intuitive interactions, and non-verbal communication. Findings highlight the importance of structured workshop discussions, the use of visual aids, and the creation of an inclusive environment to facilitate participation by PLwD. Participants reported that the robot provided a calming experience and demonstrated potential for use in care settings. However, some PLwD expressed hesitation in sharing opinions when caregivers were present, suggesting the need for separate workshops to foster uninhibited feedback. This study provides a model for integrating intended users into the design process, offering insights for researchers developing assistive technologies in dementia care.

